# Inflammation biomarkers and delirium in critically ill patients: new insights?

**DOI:** 10.1186/cc13930

**Published:** 2014-06-18

**Authors:** Shokoufeh Cheheili Sobbi, Mark van den Boogaard

**Affiliations:** 1 Department of Intensive Care Medicine, Radboudumc, Internal Post: 710 - Research IC, Nijmegen, P.O. 9101, 6500HB, the Netherlands

## Abstract

The pathophysiological mechanism of the serious and frequently occurring disorder delirium is poorly understood. Inflammation and sepsis are known risk factors for ICU delirium and therefore these patients are highly susceptible to delirium. Several studies have been performed to determine which cytokines are most associated with delirium but the results are inconclusive. Also, new biomarkers associated with brain dysfunction and cognitive impairment are still recognized and need to be studied to determine their relation with delirium. In this commentary we address some limitations concerning an interesting new study that warrants directions for future studies.

## 

We read with interest the study by Ritter and colleagues in which the relationship between inflammatory biomarkers and the development of delirium was investigated [1]. While delirium in critically ill patients is recognized as a major problem associated with deleterious outcome, the pathophysiology is still poorly understood. Apart from a role in further unraveling pathophysiological pathways in delirium, biomarkers could possibly also be used as diagnostic or prognostic disease markers. This task might prove to be difficult, as delirium is a multifactorial disorder and thus several pathways are probably involved in its development [2]. Studying the underlying mechanism of delirium in a relatively homogeneous study population, as Ritter and colleagues did in 78 inflamed patients [1], could minimize the interaction between different pathways. Patients with systemic inflammation or sepsis are highly vulnerable to developing brain dysfunction and delirium, also defined as sepsis-associated delirium or sepsis-associated encephalopathy [3], and could therefore serve this purpose.

Ritter and colleagues studied TNFα, soluble TNF receptor (STNFR)-1, STNFR2, IL-1β, IL-6, IL-10 and adiponectin in systemic inflamed patients in relation to delirium [1]. In their prospective cohort study they found significant associations between STNFR1, STNFR2, IL-1β and adiponectin concentrations and the development of delirium.

We previously found that delirium was associated with IL-8 (odds ratio, 9.0; 95% confidence interval, 1.8 to 44.0) and IL-10 (odds ratio, 2.6; 95% confidence interval, 1.1 to 5.9) but not with TNFα in inflamed ICU patients [4]. In contrast, Ritter and colleagues found no association between IL-10 and delirium. This discrepancy might be explained by differences in study design: Ritter and colleagues collected blood samples within 12 hours of ICU admission, while in our study blood was drawn within 24 hours after the onset of delirium regardless of ICU length of stay. In view of the changes over time in the concentration of cytokines and the development of delirium, serial determinations of circulating inflammatory markers and the relationship with the development of delirium would be of great interest, but this has not been carried out in ICU patients up to now.

Surprisingly, Ritter and colleagues found no differences in several patient characteristics such as age, severity of illness scores, and duration of mechanical ventilation, but also not in the presence of sepsis, between delirious and nondelirious ICU patients, while these characteristics are clearly recognized as risk factors for delirium [5-7]. A possible explanation for why they did not find these differences could be the frequency of delirium assessment. Due to its fluctuating course, delirium can be missed when patients are assessed with the Confusion Assessment Method for the Intensive Care Unit only twice a day – especially when it is recognized that on average 20% of delirium is missed when using the Confusion Assessment Method for the Intensive Care Unit [8]. This fact may also explain the observed relatively low delirium incidence (39.7%) in these highly susceptible patients suffering from systemic inflammation.

Another interesting and very relevant point is that Ritter and colleagues also studied, besides the common proinflammatory and anti-inflammatory cytokines, the hormone adiponectin. Adiponectin was recently determined to interact with the brain [9] and to play a role in neuroprotection and energy expenditure. Levels of adiponectin are elevated in critically ill patients [10] and even higher in delirious ICU patients, as Ritter and colleagues determined [1]. These authors are the first to determine an association between adiponectin levels (adjusted for weight) and delirium.

Although not a primary study aim, Ritter and colleagues also determined the accuracy of the prediction of delirium using these biomarkers. Even though IL-β, STNFR1, STNFR2 and adiponectin individually predict delirium moderately well (area under the receiver operating characteristics curve: 0.70 to 0.84), the authors did not mention the overall area under the receiver operating characteristics curve of the model using multivariate regression analysis. Unfortunately the sample size in their study did not allow inclusion of other relevant risk factors. In daily clinical practice, therefore, using a delirium prediction model specifically developed for prediction based on clinical risk factors appears better [11]. Despite these shortcomings, their study was well designed and their findings are very relevant to further progress this field.

Currently, the role of biomarkers as Ritter and colleagues determined is, although relevant, still limited for daily clinical practice. Serial measurements of inflammatory markers in ICU patients and further research into the role of adiponectin in the development of delirium warrant further investigation in future studies.

## Abbreviations

IL: Interleukin; STNFR: Soluble tumor necrosis factor receptor; TNF: Tumor necrosis factor.

## Competing interests

The authors declare that they have no competing interests.
